# Supplementary figures and images for: Modeling 5-FU-Induced Chemotherapy Selection of a Drug-Resistant Cancer Stem Cell Subpopulation
Source: Curr Oncol. 2024 Feb 25;31(3):1221–34. doi: 10.3390/curroncol31030091 (PMC10968802; doi:10.3390/curroncol31030091)

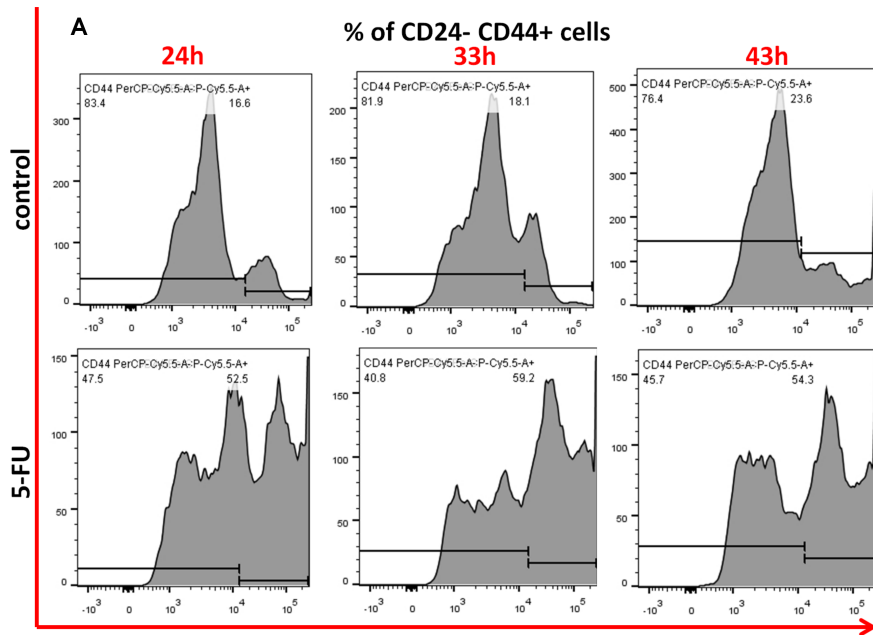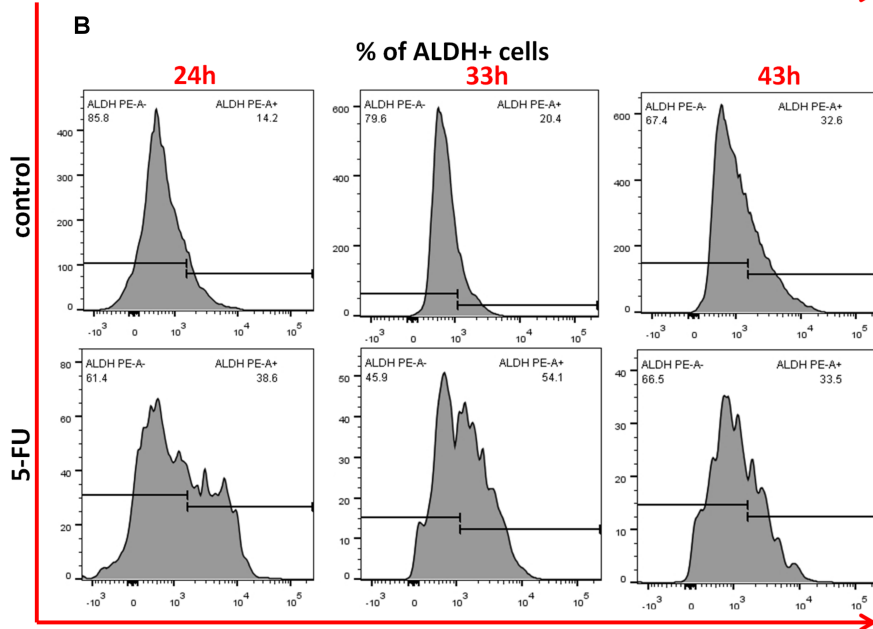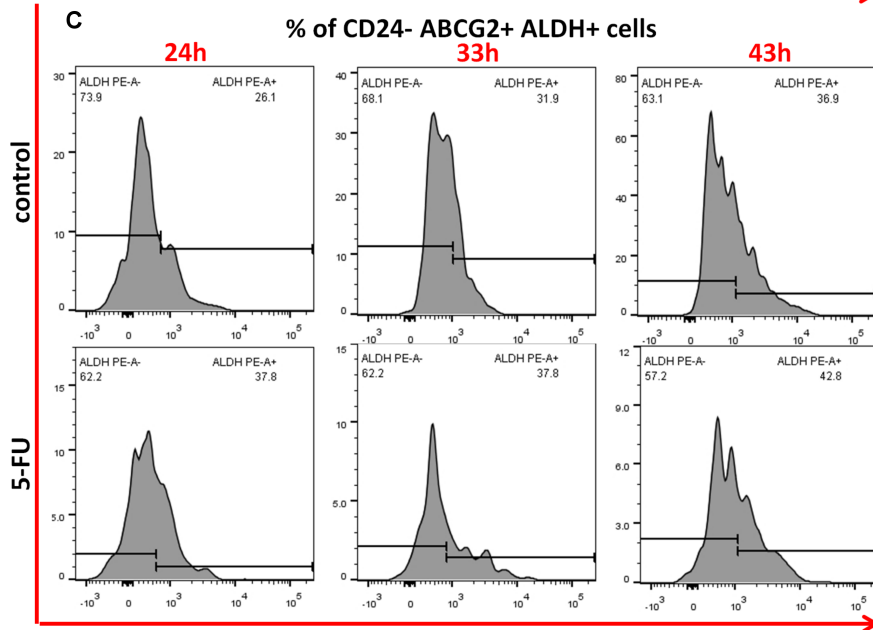

Supplement: Supplementary file 1 [file curroncol-31-00091-s001.zip › S01.pdf]

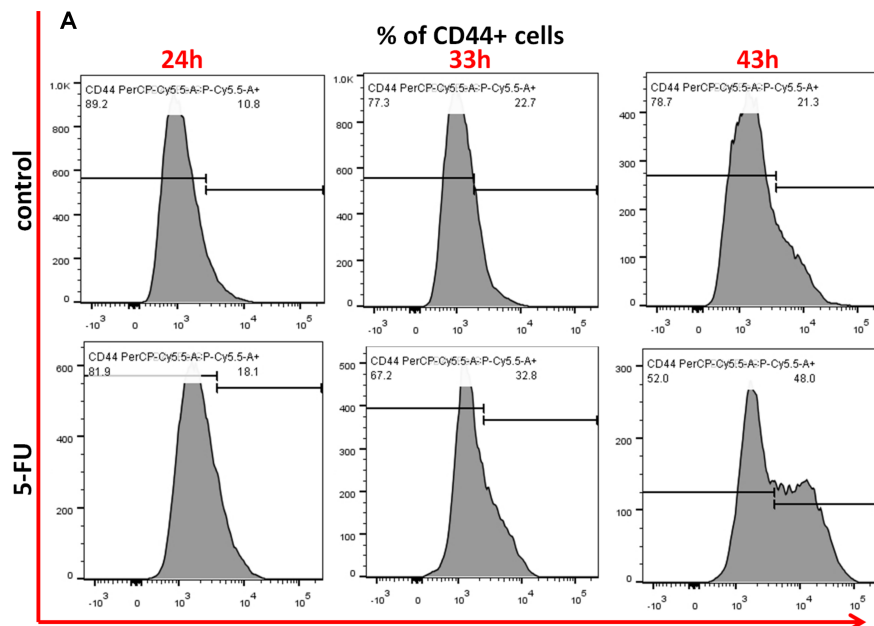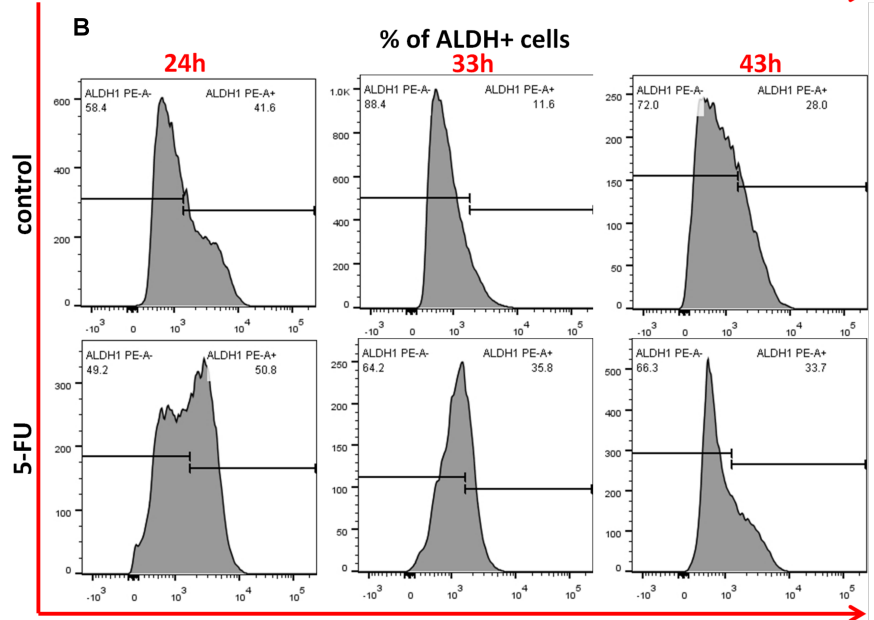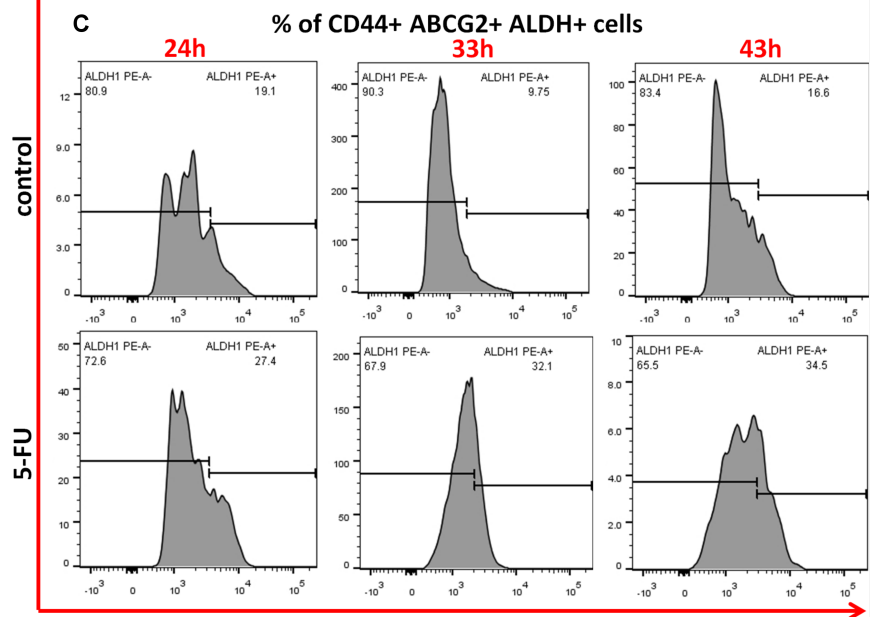

Supplement: Supplementary file 1 [file curroncol-31-00091-s001.zip › S02.pdf]

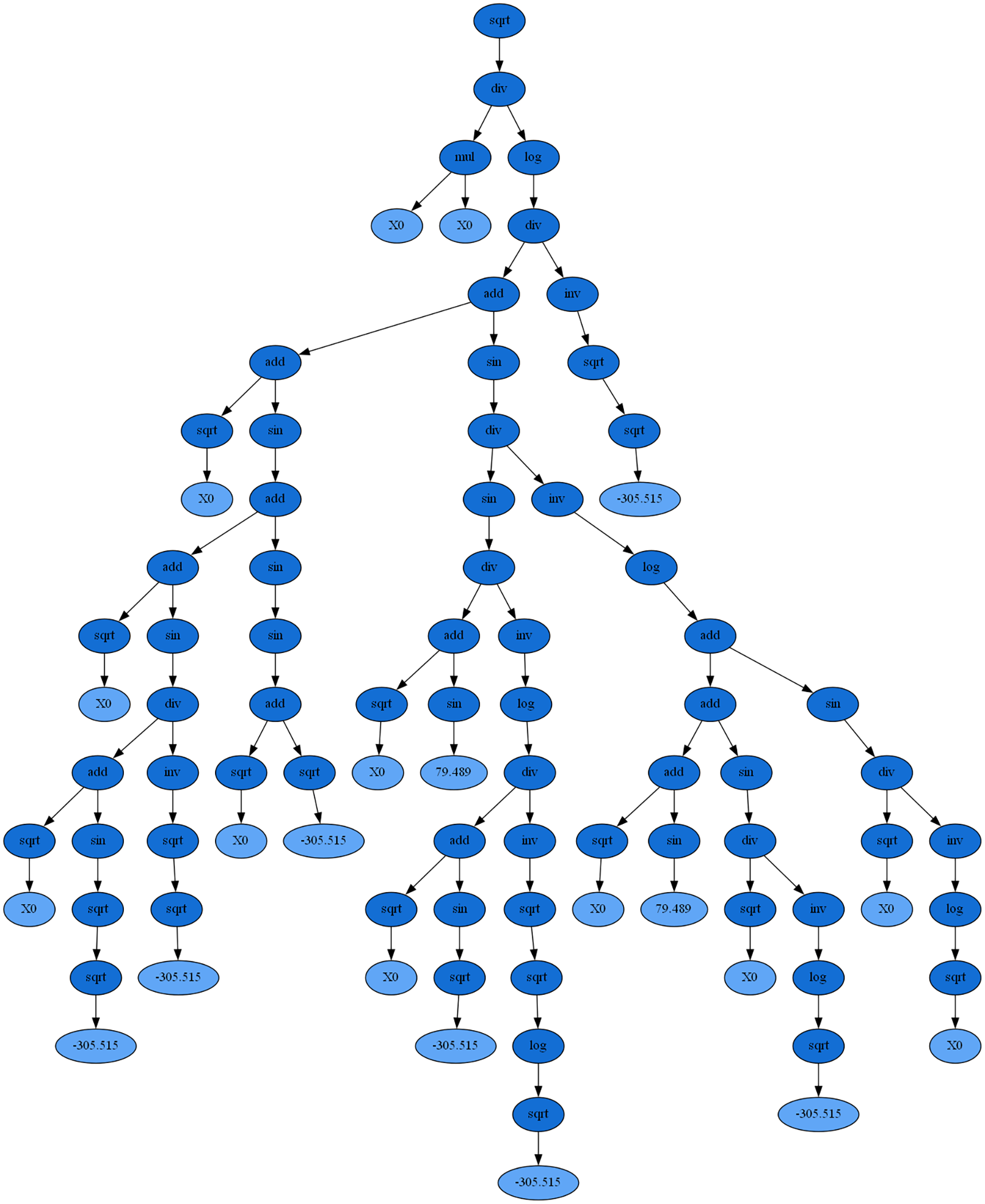

Supplement: Supplementary file 1 [file curroncol-31-00091-s001.zip › S03.tif]

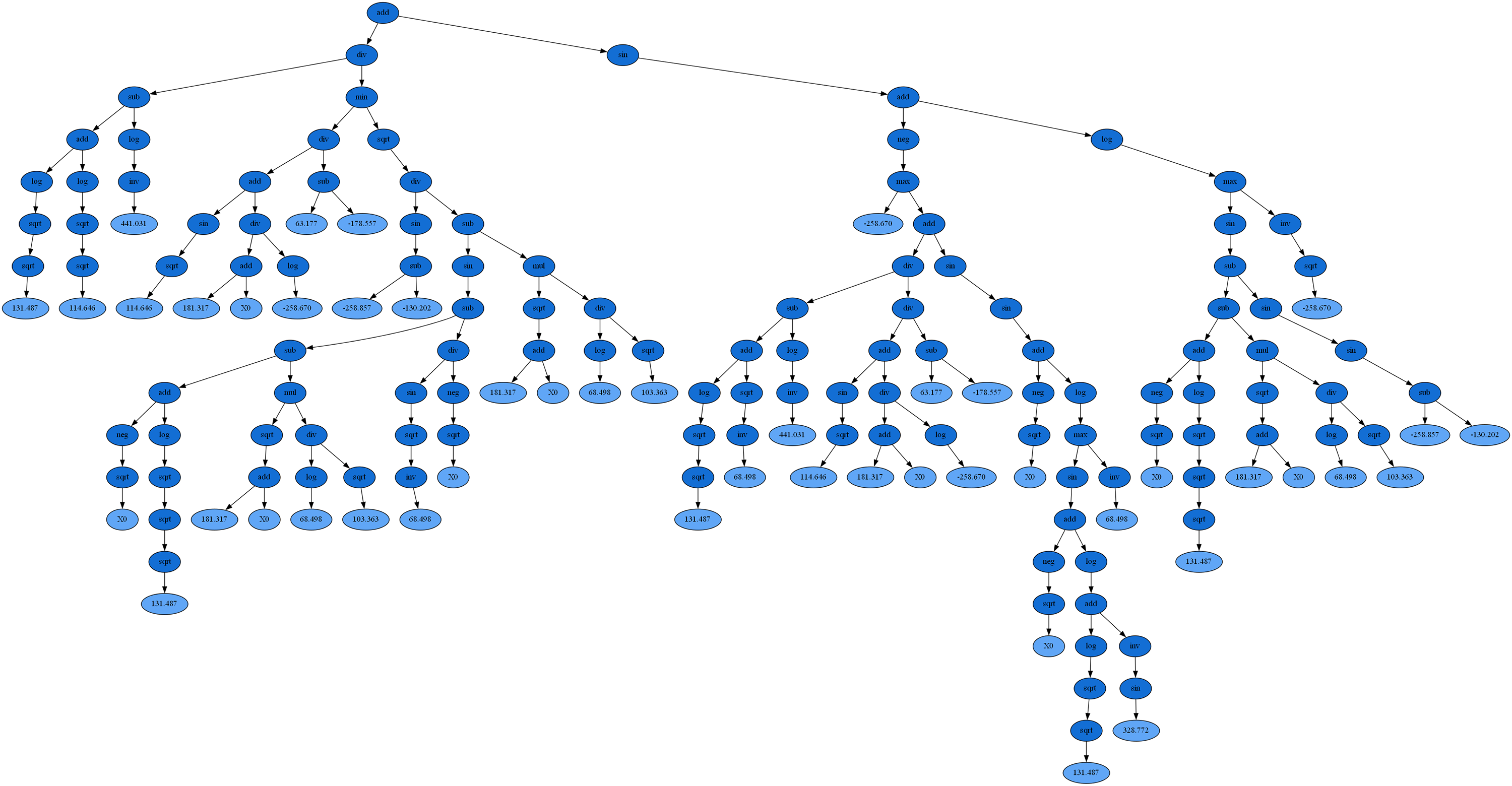

Supplement: Supplementary file 1 [file curroncol-31-00091-s001.zip › S04.tif]

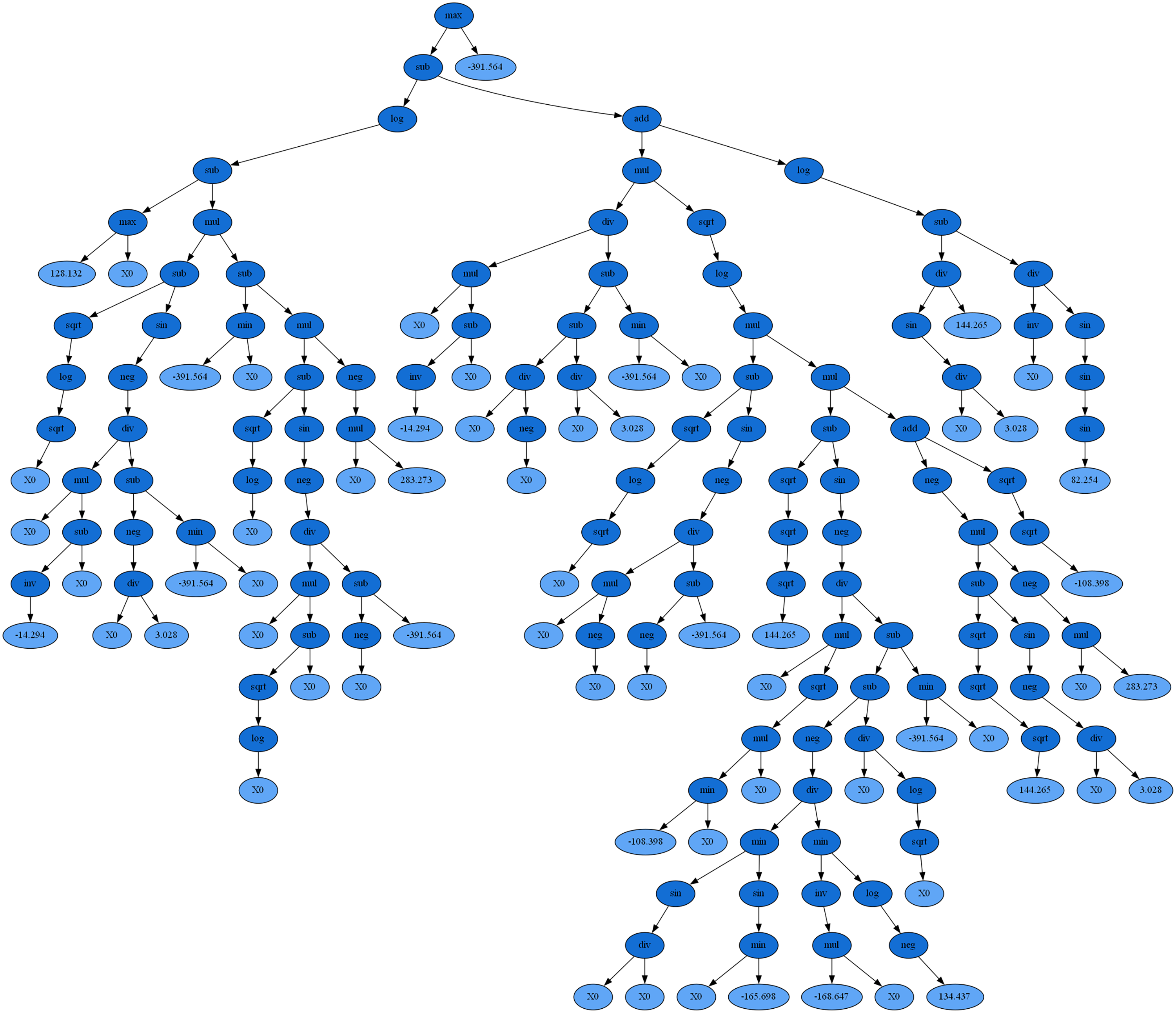

Supplement: Supplementary file 1 [file curroncol-31-00091-s001.zip › S05.tif]

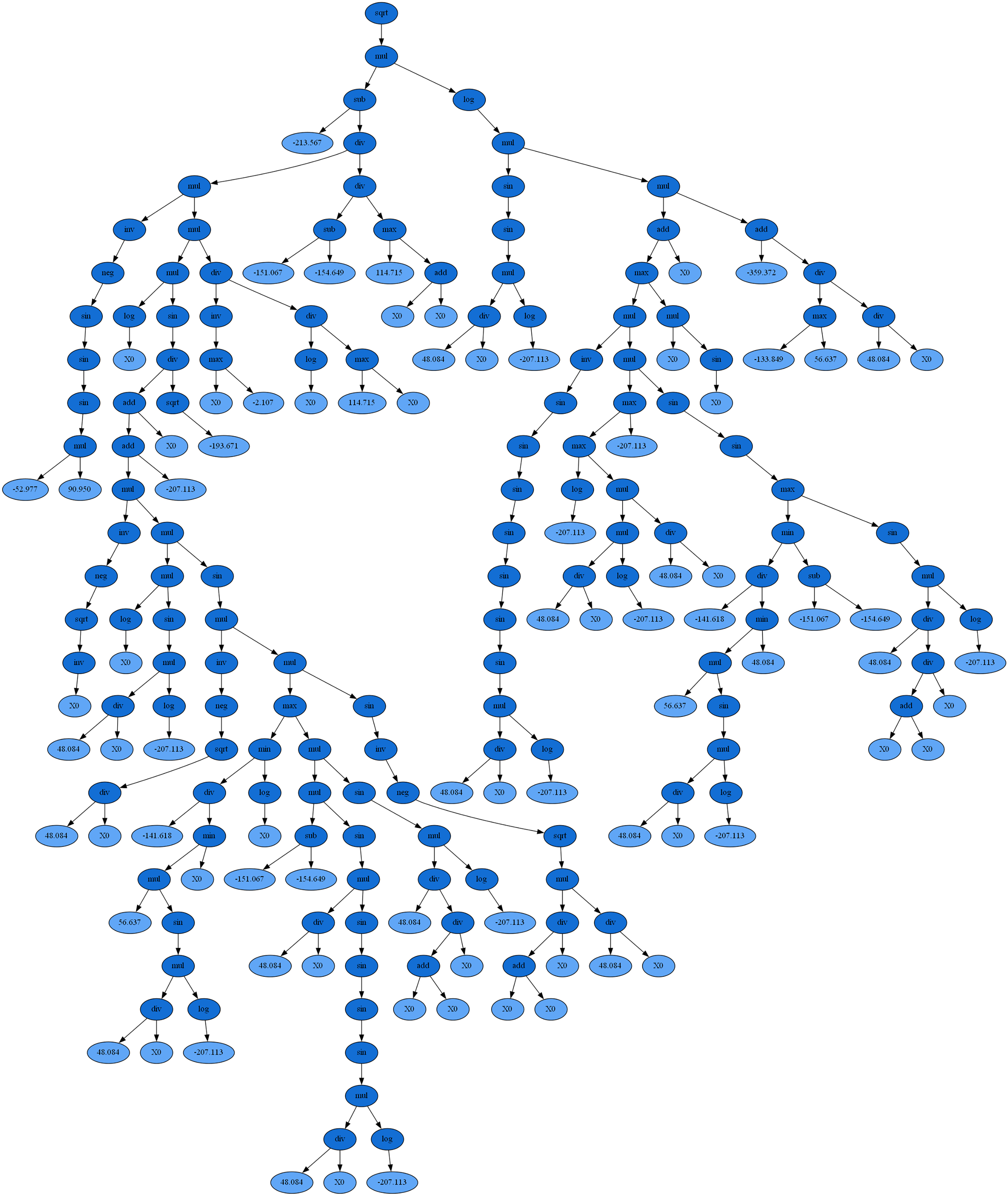

Supplement: Supplementary file 1 [file curroncol-31-00091-s001.zip › S06.tif]

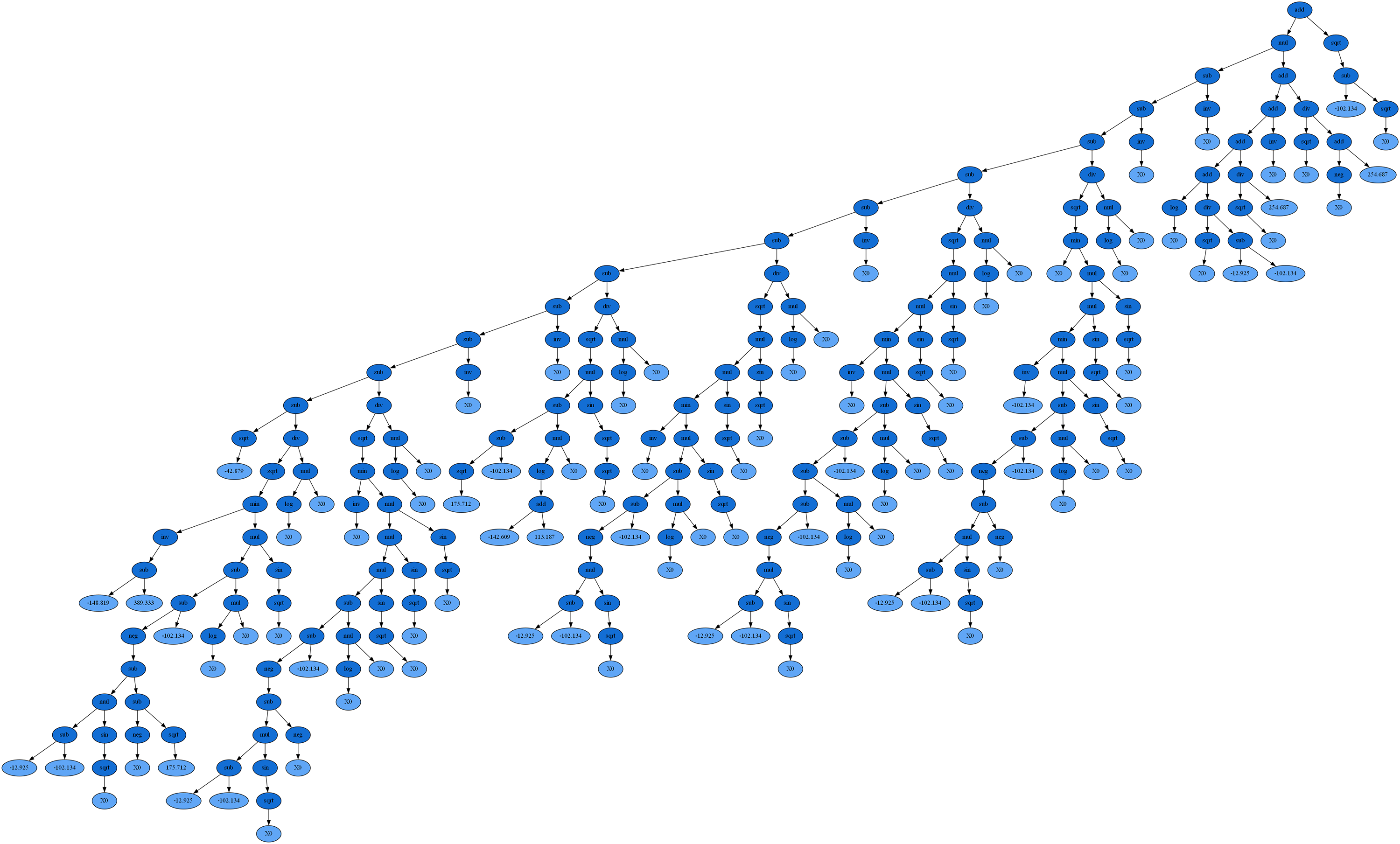

Supplement: Supplementary file 1 [file curroncol-31-00091-s001.zip › S07.tif]

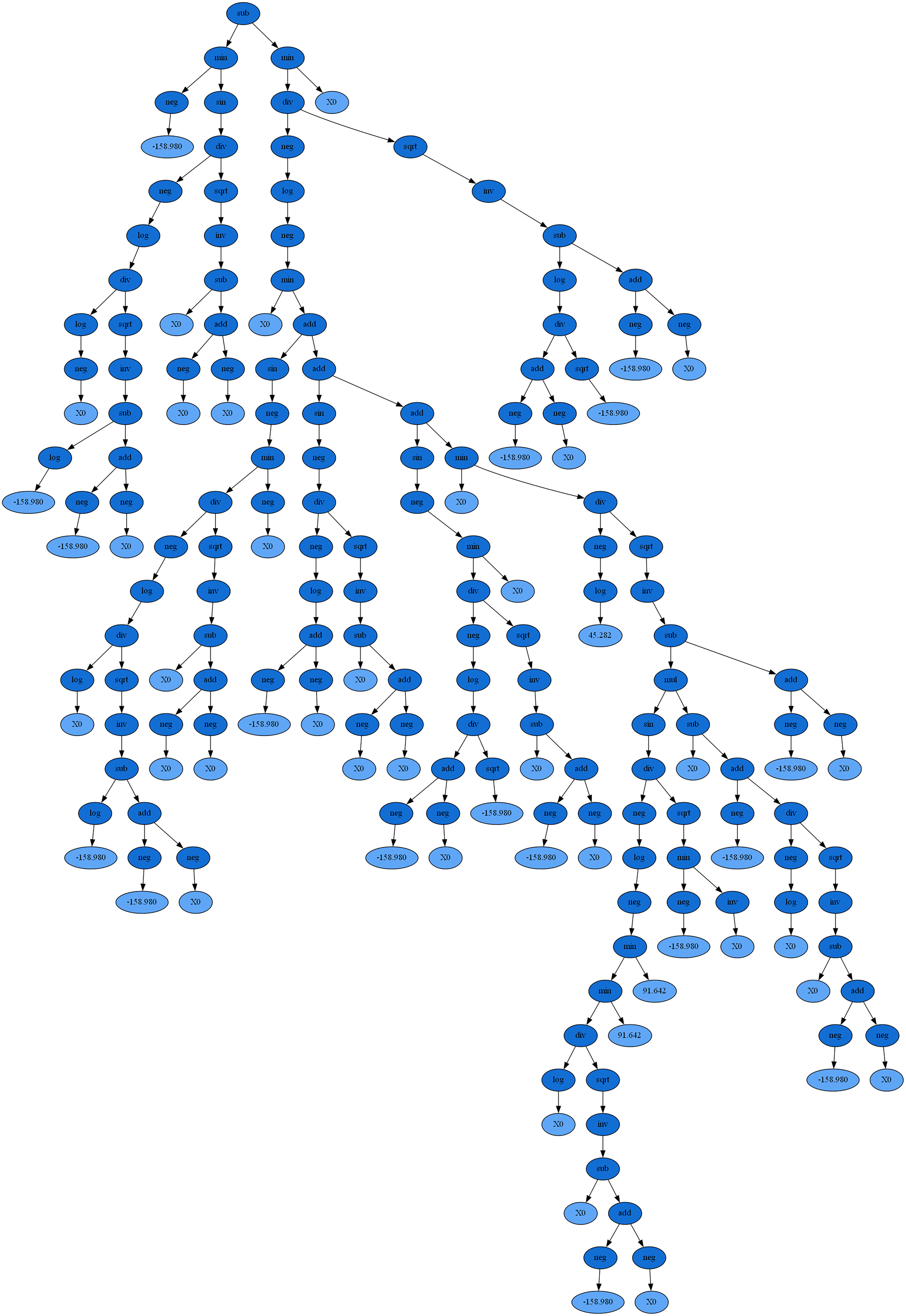

Supplement: Supplementary file 1 [file curroncol-31-00091-s001.zip › S08.tif]

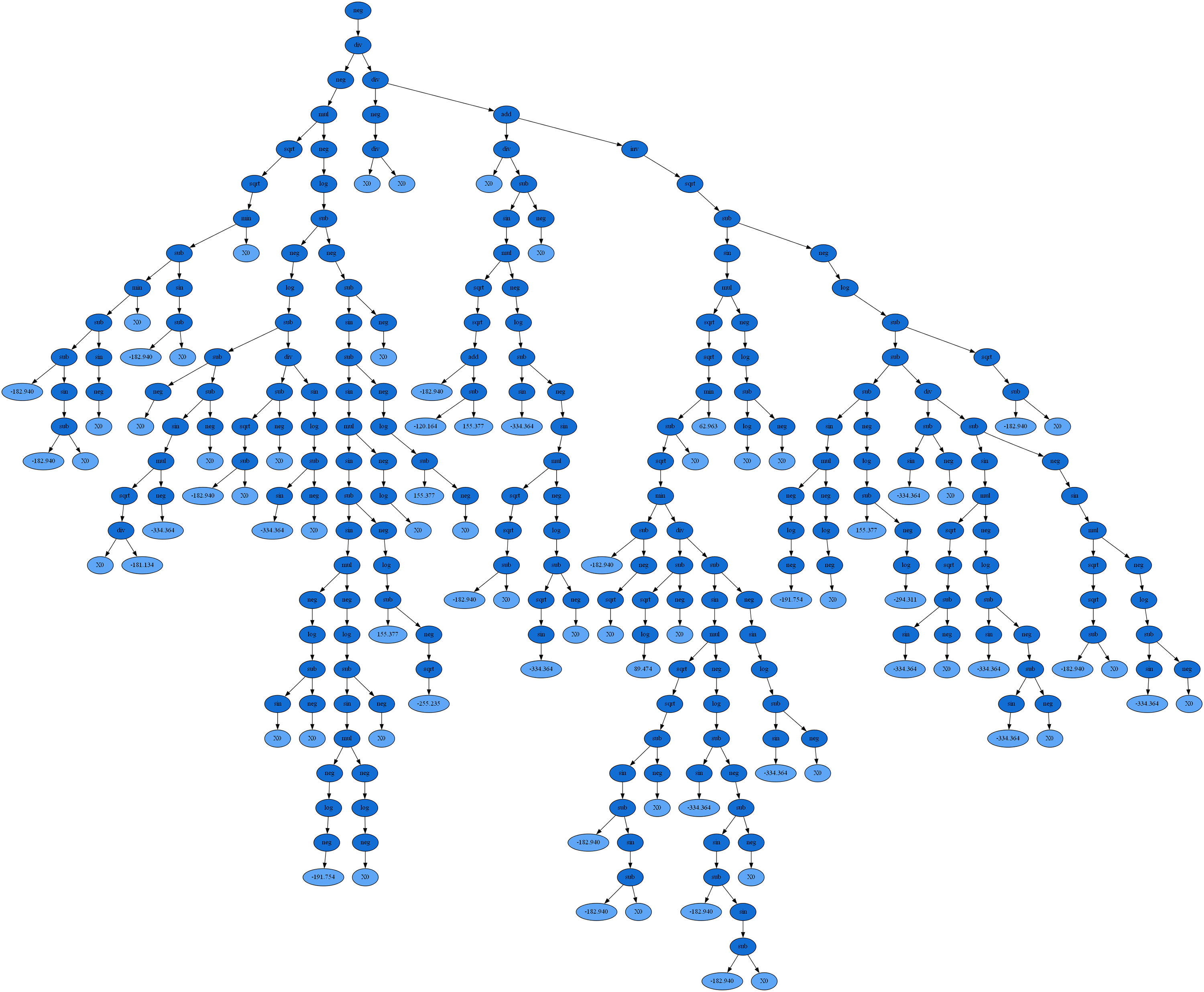

Supplement: Supplementary file 1 [file curroncol-31-00091-s001.zip › S09.tif]

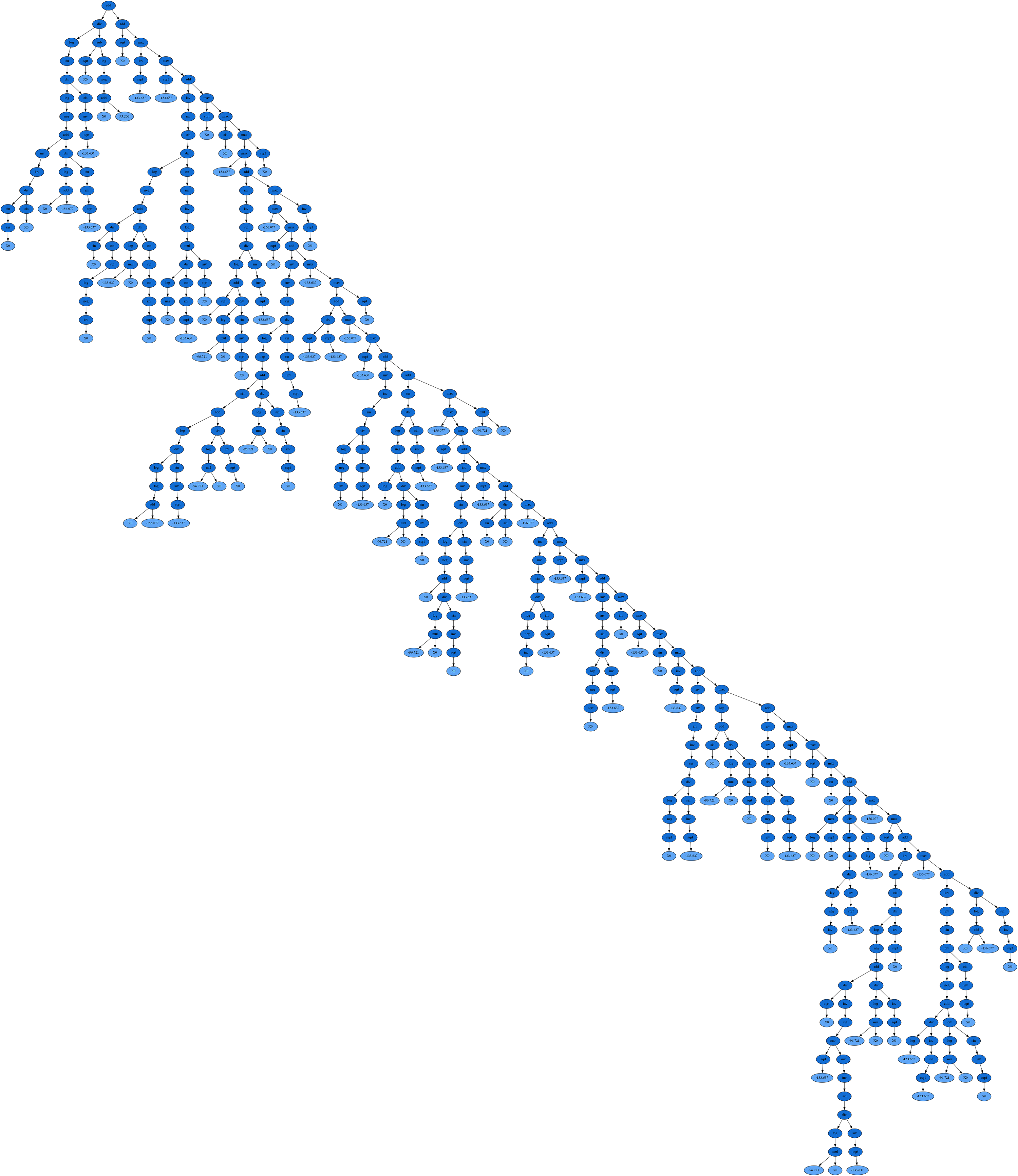

Supplement: Supplementary file 1 [file curroncol-31-00091-s001.zip › S10.tif]

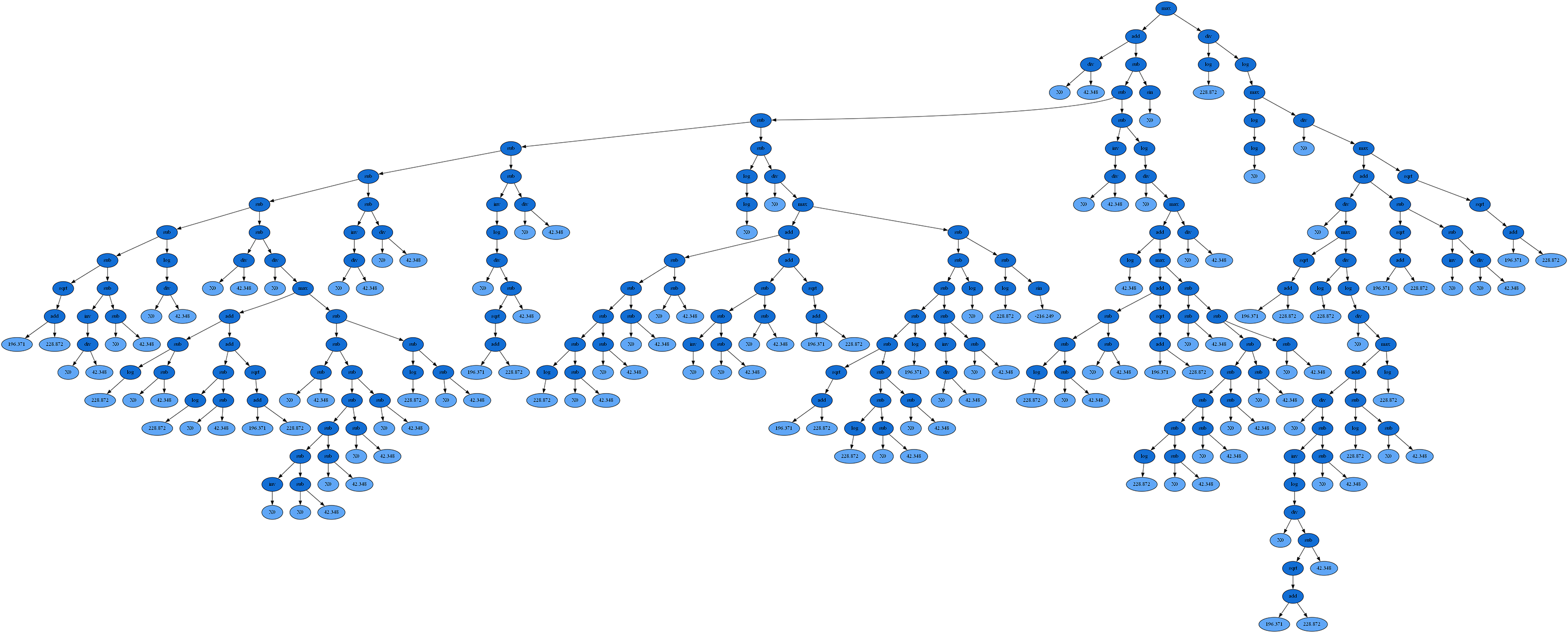

Supplement: Supplementary file 1 [file curroncol-31-00091-s001.zip › S11.tif]

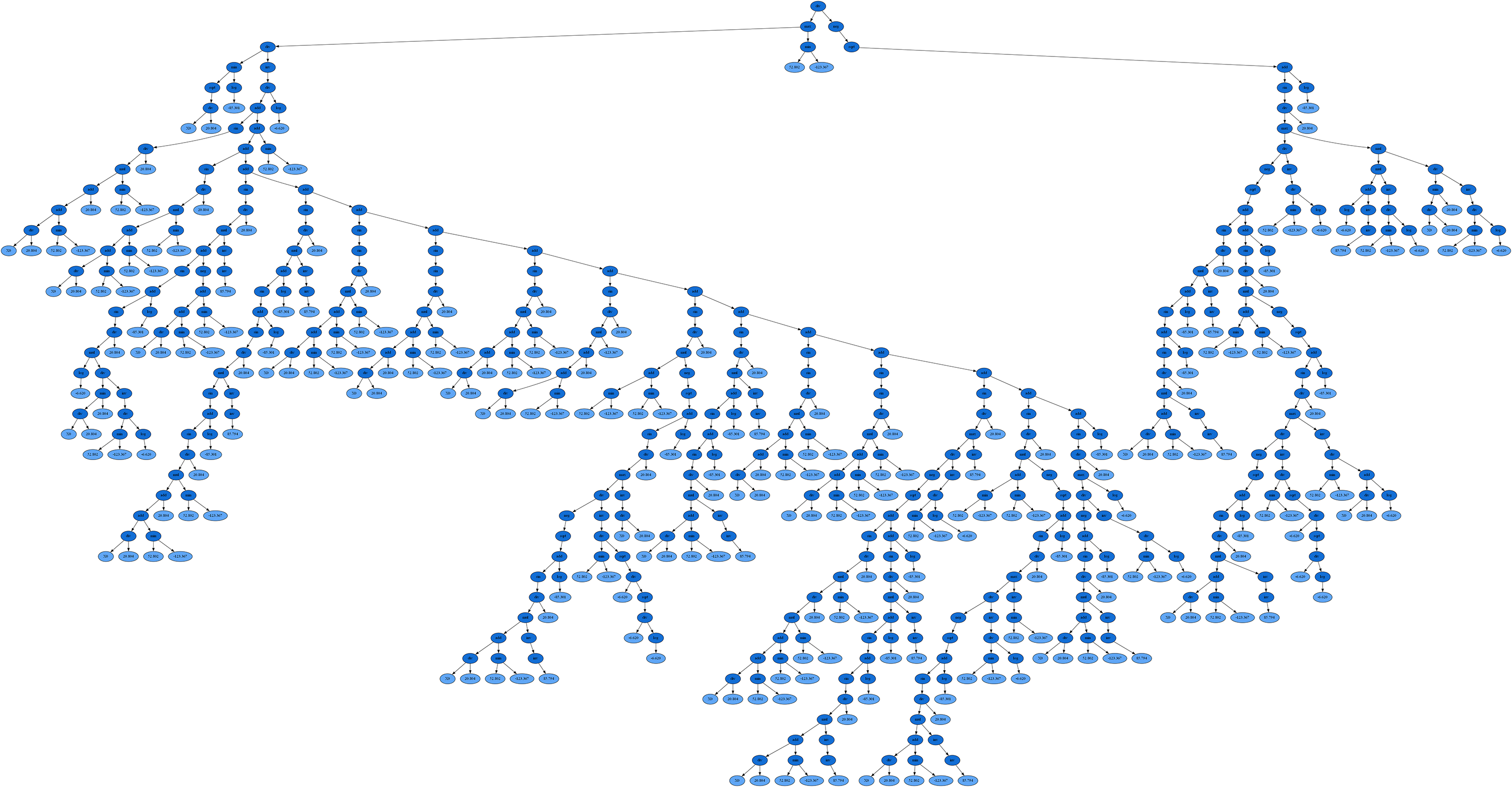

Supplement: Supplementary file 1 [file curroncol-31-00091-s001.zip › S12.tif]

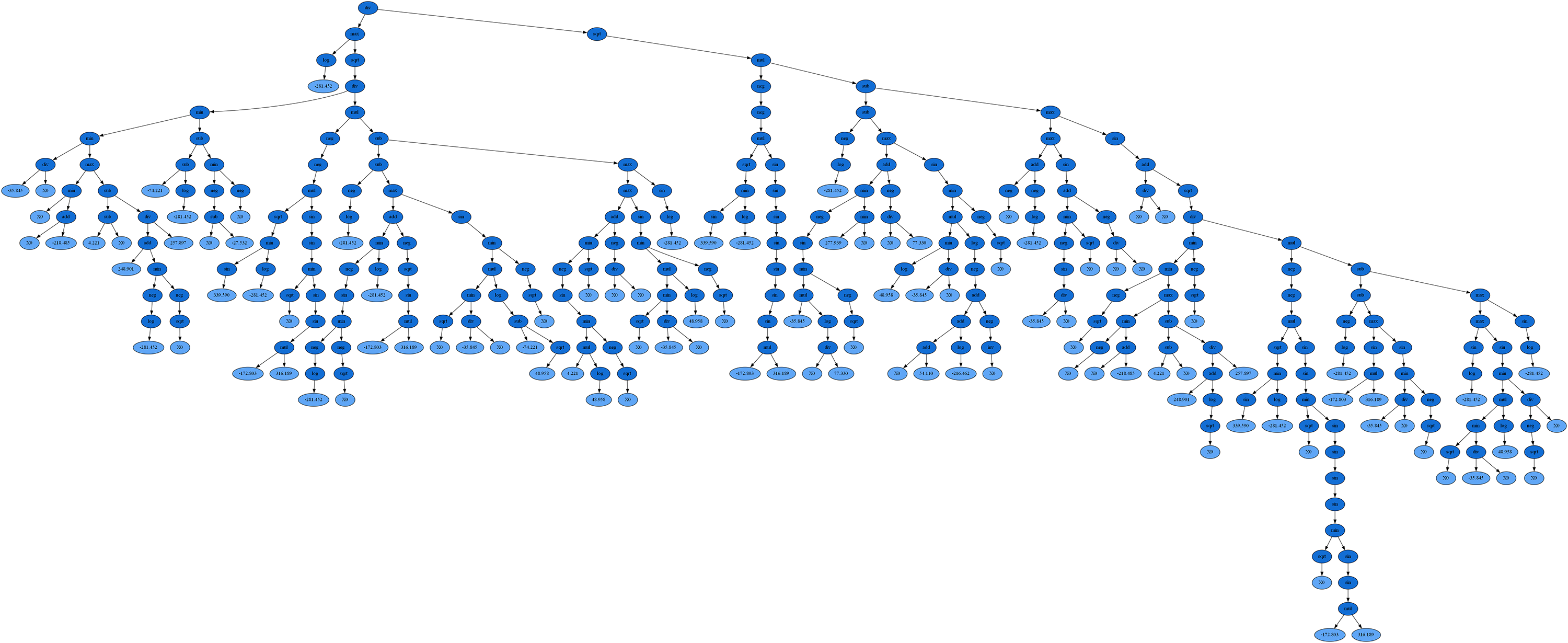

Supplement: Supplementary file 1 [file curroncol-31-00091-s001.zip › S13.tif]

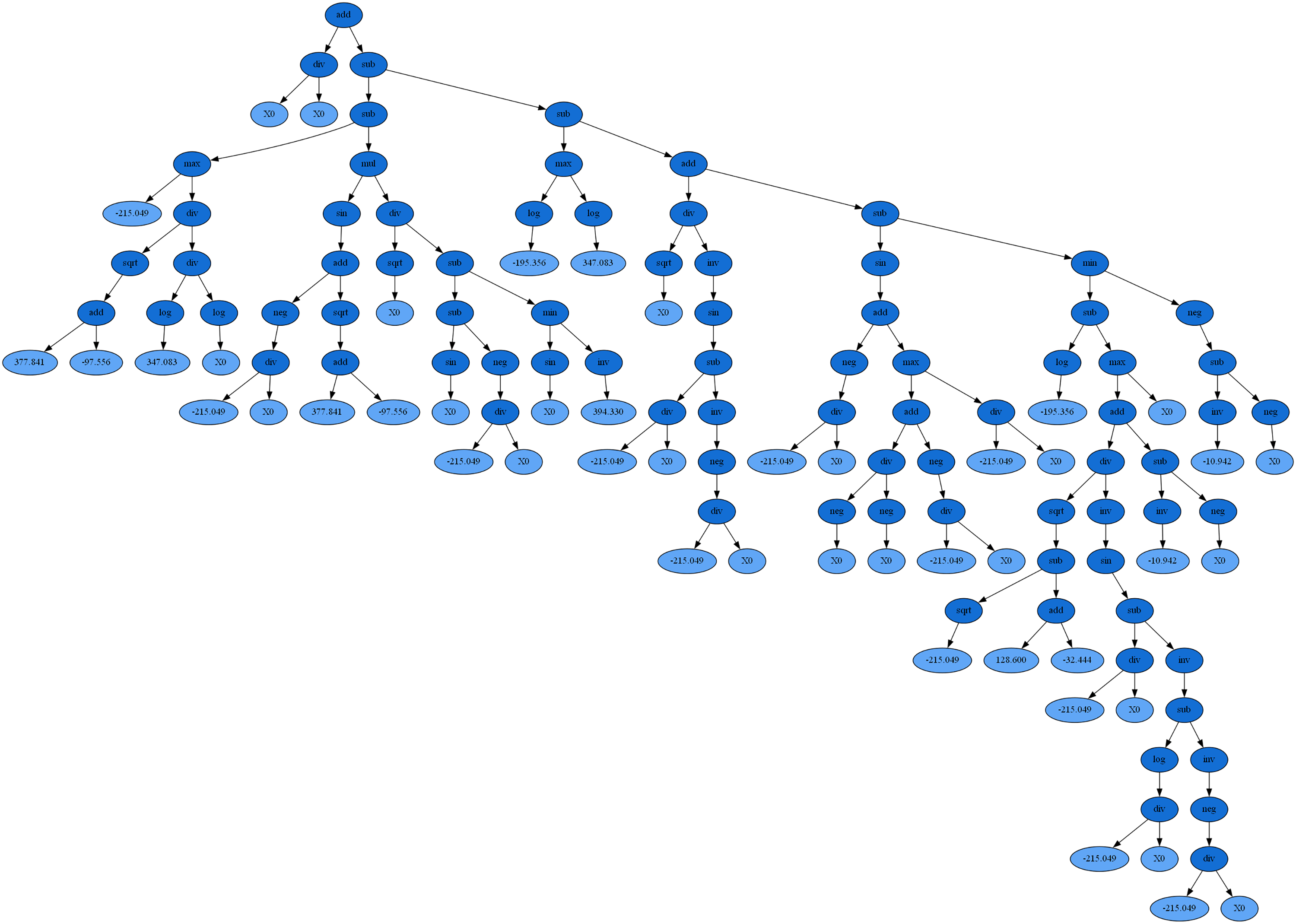

Supplement: Supplementary file 1 [file curroncol-31-00091-s001.zip › S14.tif]

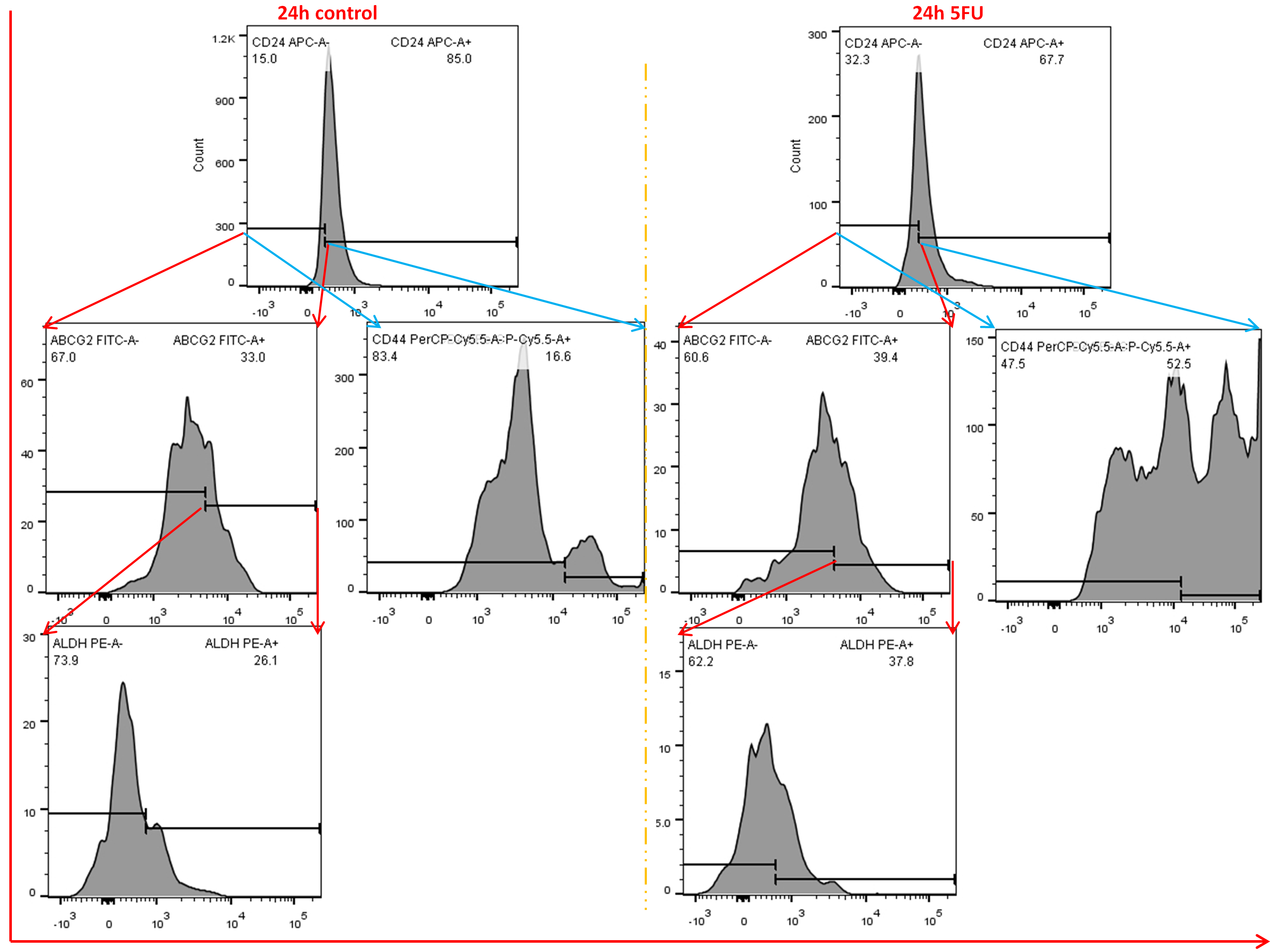

Supplement: Supplementary file 1 [file curroncol-31-00091-s001.zip › S15 MDA Control + 5FU CD24- Abcg2+ ALDH+ i CD24- CD44+ gating strategy.tif]

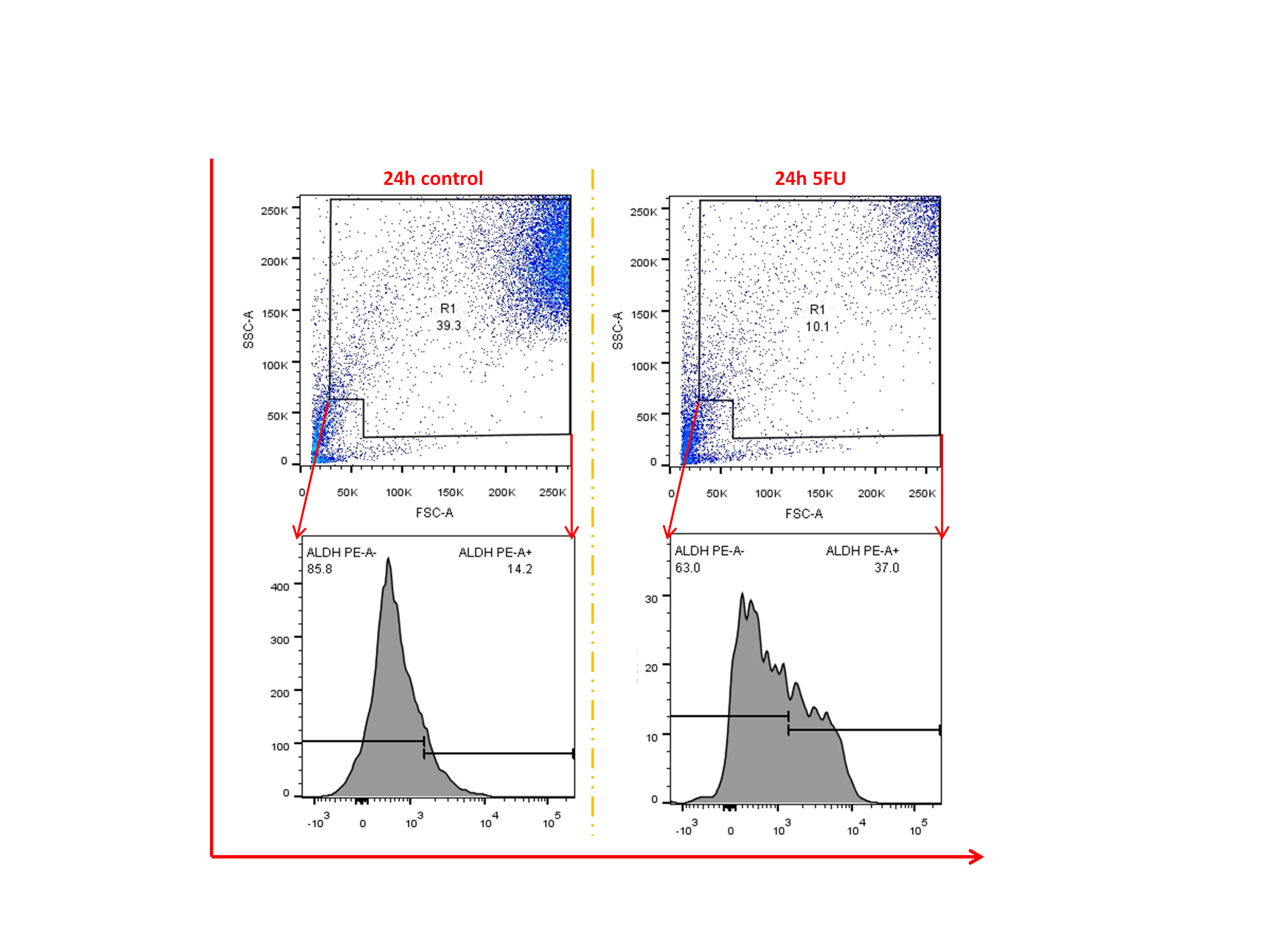

Supplement: Supplementary file 1 [file curroncol-31-00091-s001.zip › S16 MDA MB Control + 5FU ALDH+ rep plots.tiff]

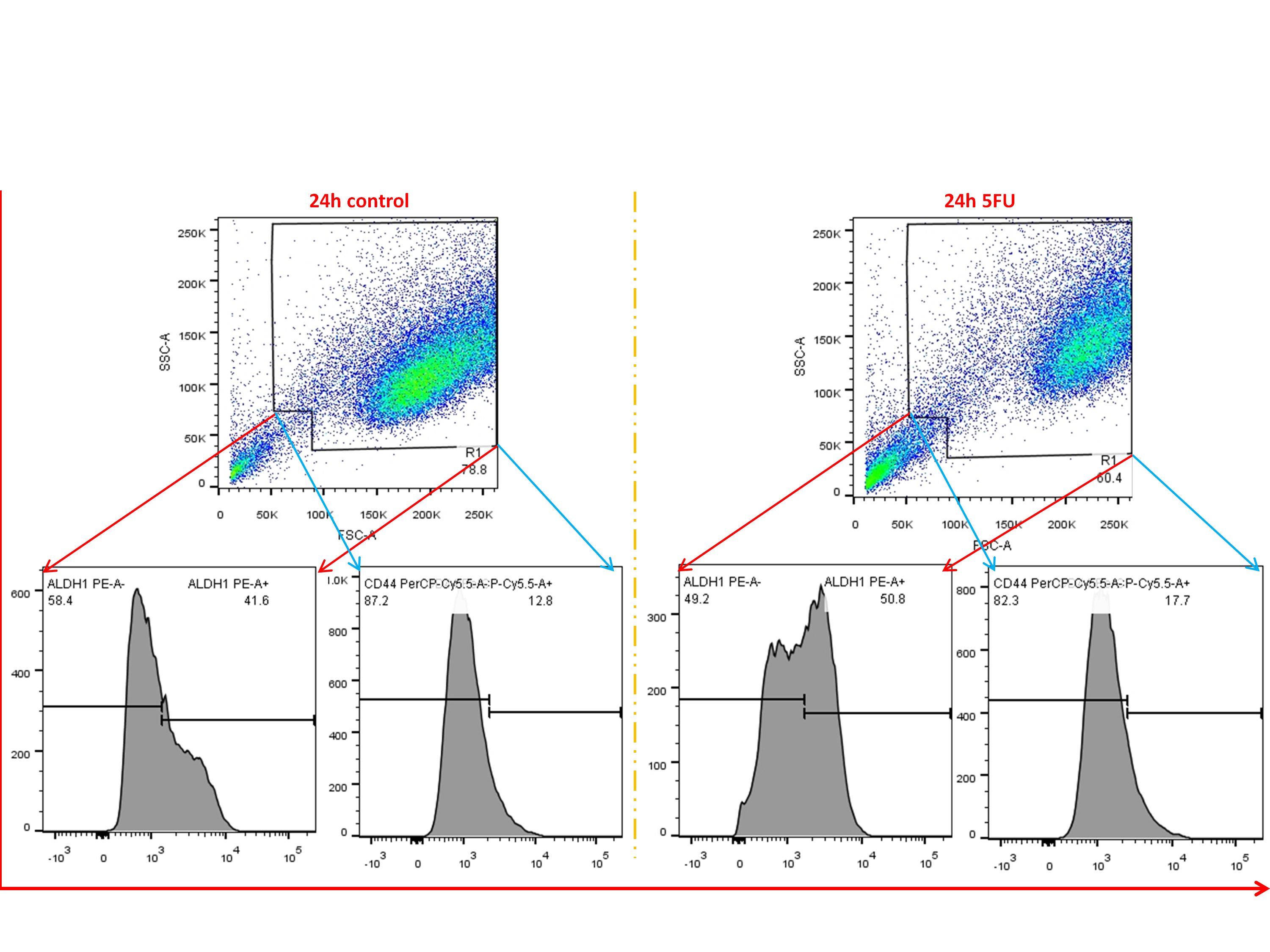

Supplement: Supplementary file 1 [file curroncol-31-00091-s001.zip › S17 HCT control + 5FU CD44+ i ALDH1+ gating strategy.tif]

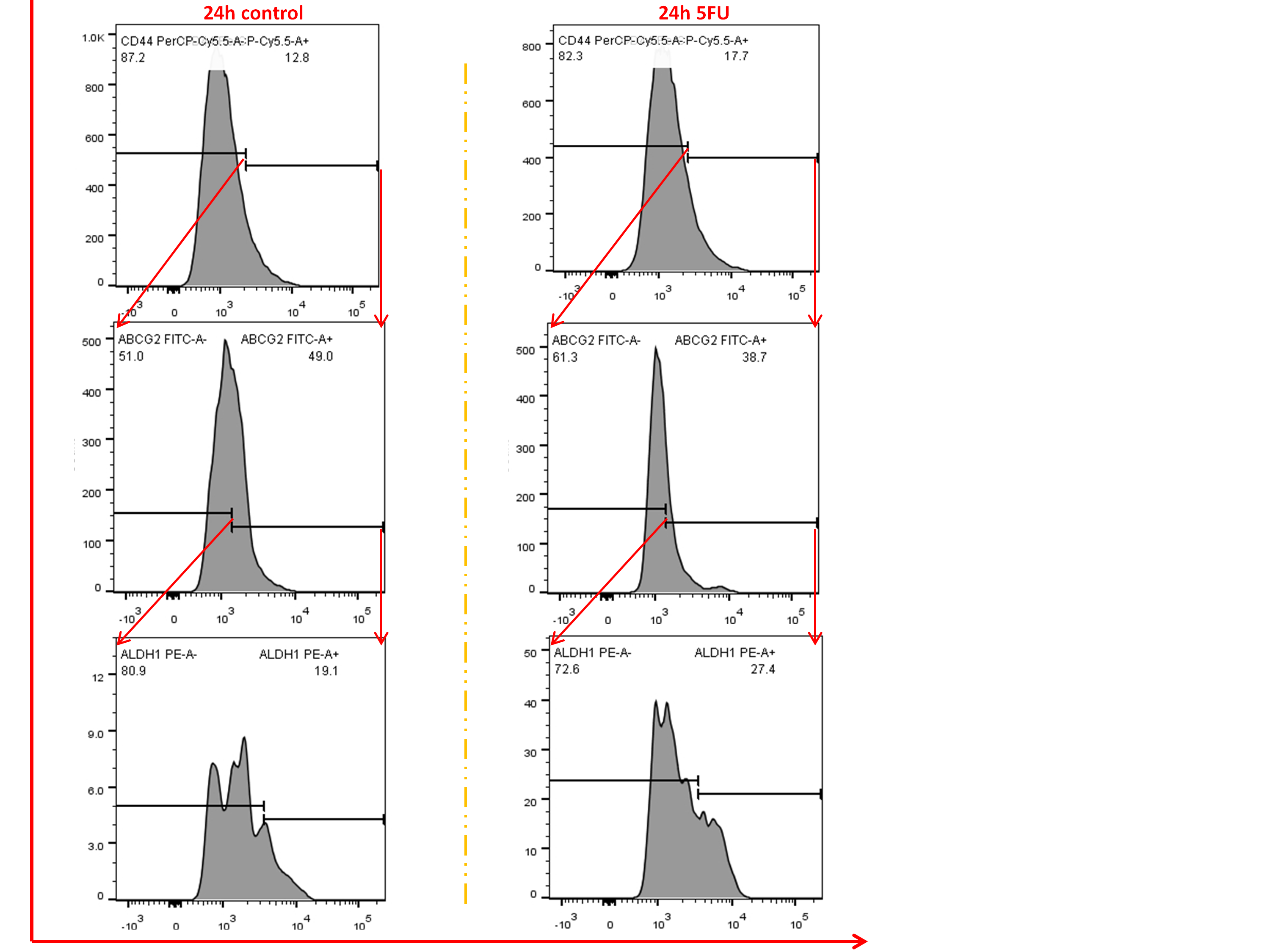

Supplement: Supplementary file 1 [file curroncol-31-00091-s001.zip › S18 HCT control + 5FU CD44+ ABCG2+ ALDH1+ gating strategy.tif]
